# COVID-19 Infection-Related Coagulopathy and Viscoelastic Methods: A Paradigm for Their Clinical Utility in Critical Illness

**DOI:** 10.3390/diagnostics10100817

**Published:** 2020-10-14

**Authors:** Argirios E. Tsantes, Andreas G. Tsantes, Styliani I. Kokoris, Stefanos Bonovas, Frantzeska Frantzeskaki, Iraklis Tsangaris, Petros Kopterides

**Affiliations:** 1 Laboratory of Hematology and Blood Bank Unit, “Attikon” University Hospital, School of Medicine, National and Kapodistrian University of Athens, 1 Rimini Str., 12462 Athens, Greece; andreas.tsantes@yahoo.com (A.G.T.); stelkok19@gmail.com (S.I.K.); 2 Department of Biomedical Sciences, Humanitas University, Via Rita Levi Montalcini 4, Pieve Emanuele, 20090 Milan, Italy; sbonovas@gmail.com; 3 Humanitas Clinical and Research Center, IRCCS, Via Manzoni 56, Rozzano, 20089 Milan, Italy; 4 Second Department of Critical Care, “Attikon” University Hospital, Medical School, National and Kapodistrian University of Athens, 12462 Athens, Greece; ffrantzeska@gmail.com (F.F.); itsagkaris@med.uoa.gr (I.T.); 5 Intensive Care Unit, Excela Health Westmoreland Hospital, Greensburg, PA 15601, USA; pkopterides@gmail.com

**Keywords:** blood coagulation disorder, COVID-19, viscoelastic methods, thrombosis

## Abstract

Hypercoagulability and thrombosis remain a challenge to diagnose and treat in severe COVID-19 infection. The ability of conventional global coagulation tests to accurately reflect in vivo hypo- or hypercoagulability is questioned. The currently available evidence suggests that markedly increased D-dimers can be used in identifying COVID-19 patients who may need intensive care unit (ICU) admission and close monitoring or not. Viscoelastic methods (VMs), like thromboelastography (TEG) and rotational thromboelastometry (ROTEM), estimate the dynamics of blood coagulation. The evaluation of coagulopathy by VMs in severe COVID-19 infection seems an increasingly attractive option. Available evidence supports that COVID-19 patients with acute respiratory failure suffer from severe hypercoagulability rather than consumptive coagulopathy often associated with fibrinolysis shutdown. However, the variability in definitions of both the procoagulant profile and the clinical outcome assessment, in parallel with the small sample sizes in most of these studies, do not allow the establishment of a clear association between the hypercoagulable state and thrombotic events. VMs can effectively provide insight into the pathophysiology of coagulopathy, detecting the presence of hypercoagulability in critically ill COVID-19 patients. However, it remains unknown whether the degree of coagulopathy can be used in order to predict the outcome, establish a diagnosis or guide anticoagulant therapy.

## 1. Introduction

Critically ill coronavirus disease 2019 (COVID-19) patients are at risk of developing not only hypoxia and excessive inflammation but, also, frequent thrombotic manifestations [1]. Hypercoagulability and thrombosis remain a challenge to diagnose and treat in severe COVID-19 infections [2].

In sepsis, the disturbance between components of the coagulation and fibrinolytic system leads to a variable clinical picture, tilting from an initial hypercoagulability towards a subsequent hypocoagulable disease state, depending on the phase of septic coagulopathy [3]. Bleeding complications are rare in severe COVID-19 patients, suggesting that disseminated intravascular coagulation (DIC) and consumption coagulopathy are not common complications of COVID-19, while pulmonary microthrombosis seems to be partially related with the pathophysiological mechanism of COVID-19-related acute respiratory distress syndrome (ARDS) [4].

COVID-19-related proinflammatory cytokines induce an endothelial injury, leading to primary hemostasis activation and the overexpression of the tissue factor [2]. The resultant enhanced procoagulant activity, in association with suppressed plasmin activity by the reduced urokinase-type plasminogen activator and increased plasminogen activator inhibitor-1 [5], contributes to fibrin deposition, forming localized/disseminated microthrombi and worse clinical outcomes [6,7].

The recommended management of COVID-19 coagulopathy is based on the currently available evidence that markedly increased D-dimer levels can be used as a predictor of the development of ARDS, the need for admission in intensive care units (ICU) and death. Increased D-dimer levels in COVID-19 patients have been attributed to excess thrombin generation secondary to endothelial activation induced by the infectious stimuli, severe hypoxemia and local pulmonary microthrombosis [8].

In this context, the International Society of Thrombosis and Haemostasis (ISTH) recommends measuring the D-dimers, prothrombin time and platelet count (decreasing order of importance) in all patients who present with COVID-19 infection [9], in order to help in identifying those who may need ICU admission and close monitoring or not.

The ability of conventional global coagulation tests to reflect in vivo hypo- or hypercoagulability accurately is questioned [10], as these assays mirror only a part of the coagulation system and do not provide information on the full balance between coagulation and anticoagulation. On the contrary, viscoelastic methods (VMs), like thromboelastography (TEG) and rotational thromboelastometry (ROTEM), estimate the dynamics of blood coagulation from the activation of clotting factors to fibrin formation, clot stabilization and, finally, clot lysis [11]. The thromboelastogram can reveal coagulopathy, even when this is not detected by conventional coagulation tests [3]. Under these circumstances, a critical question is whether the use of VMs could be helpful to better explore coagulopathy, predict thrombotic events or guide anticoagulant therapy in this clinical setting [1]. The aim of this narrative review is to assess the utility of VMs in clinical practice to guide anticoagulant treatments or predict prognosis by summarizing and analyzing most of the relevant, recently published studies.

## 2. Methods

An electronic search on the PubMed and Scopus databases was carried out for studies published in 2019 and 2020. Our search strategy combined terms or keywords that are related to COVID-19 infection (COVID-19, etc.) with those related to the viscoelastic methods (viscoelastic method, thromboelastography, rotational thromboelastometry, etc.) for the identification of relevant articles. The reference lists of the extracted articles were also pursued for relevant studies that could be further included in the review. An inclusion and exclusion methodology was formed. Articles concerning the application of viscoelastic methods in the management of patients with COVID-19 infection were included. Exclusion criteria included studies published in a language other than English, studies with no full text available and case reports. A limitation of this study is that, given that this review is a narrative review and not a systematic review or meta-analysis, it lacks a quantitative data synthesis of the results of the included studies. However, even though this is not a systematic review, to the best of our knowledge, almost all of the prominent relevant studies have been included in this narrative review. Additionally, given the great heterogeneity of the included studies, the results of such a meta-analysis would not be of great value for the readers. In a future time, a meta-analysis of published studies with more consistent methodology would be more valuable.

## 3. Evaluation of Coagulopathy by Viscoelastic Methods in Severe COVID-19 Infection

Both TEG and TEM assess clot formation/lysis kinetics and clot strength by measuring the amount of a continuously applied rotational force, transmitted by the developing clot to an electromechanical transduction system. TEG assesses the viscoelastic properties of clotting native whole blood upon the activation of hemostasis by exogenously added triggers (kaolin). The following variables are measured: R (minutes), which is a measure of the clotting time from the point of coagulation ignition to the appearance of the clot, K (minutes) and K angle, which define the velocity of the clot formation, MA (mm), which defines the maximal amplitude of the clot and Lys-30, which defines the percentage decrease of the clot amplitude at 30 min post-MA.

A ROTEM analysis includes intrinsically-activated test using ellagic acid (INTEM), extrinsically-activated test with tissue factor (EXTEM) and fibrin-based extrinsically activated test with tissue factor and the platelet inhibitor cytochalasin D (FIBTEM) assays. The INTEM assay uses Ca^2+,^ phospholipids and ellagic acid to activate and assess coagulation through the intrinsic pathway. The tissue factor (TF) used in EXTEM assay activates and assesses the extrinsic pathway, while FIBTEM assay is applied to differentiate between thrombocytopenia and fibrin polymerization. The following parameters are assessed: the clotting time (CT, seconds), determined as the time elapsed from the start of the measurement until the formation of a clot 2 mm in amplitude; clot formation time (CFT, seconds) as the time elapsed from the end of the CT (amplitude of 2 mm) until a clot firmness of 20 mm is achieved; the amplitude recorded at 10, 20 and 30 min (A10, A20 and A30); α-angle, which is the angle between the central line (x-axis) and the tangent of the TEM tracing at the amplitude point of 2 mm, describing the kinetics of clot formation; maximum clot firmness (MCF, mm), which reflects the final strength of the clot; lysis index at 60 minutes (LI60), defined as the percentage of remaining clot stability in relation to the MCF following the 60-min observation period after CT and indicating the speed of fibrinolysis, and maximum lysis (ML), which reflects the percent decrease of the maximal amplitude over time.

Currently, coagulation point-of-care viscoelastic tests are increasingly used to characterize the coagulation profile of these patients. All of them have reported a procoagulant profile on ICU admission in the COVID-19 patients with ARDS, as indicated by the detection of increased clot strength. The findings and design characteristics of all relevant studies are presented in Table 1. In detail, the main finding of Ranucci et al., using an ultrasound-based technology that measures changes in the viscoelastic properties of whole blood, was a procoagulant profile in 16 COVID-19 ARDS patients and its progression toward normalization after the administration of increased thromboprophylaxis [12]. In their hands, viscoelastic tests showed normal values of clotting time but confirmed a clot firmness higher than normal by comparing the median values of the viscoelastic parameters with the upper limit of the normal reference ranges. 

Panigada et al. were the first who noted decreased or increased TEG variables in 24 COVID-19 ICU patients by comparing them with the mean of the respective reference ranges previously established in 40 healthy adult subjects [13]. The R and K time values were shorter, while the K angle and MA variables were higher than the mean value of the reference population in more than 50% of the COVID-19 population. It is noteworthy that Lys-30 was higher than the mean of the reference population in all the COVID-19 patients, indicating a fibrinolytic capacity with values very close to the limit of hyperfibrinolysis. Spiezia et al. evaluated coagulation abnormalities via ROTEM profiles in a group of 22 COVID-19 ICU patients with acute respiratory failure [14]. Forty-four healthy subjects served as controls. The cases showed markedly hypercoagulable profiles, as reflected by the significantly shorter CFT in the INTEM and EXTEM and higher MCF in the INTEM, EXTEM and FIBTEM assays compared to the controls. No abnormal fibrinolytic activity was found. Similarly, Wright and colleagues showed that COVID-19 ICU patients were hypercoagulable compared to the established reference ranges [15]. Of note, there was fibrinolysis shutdown, as demonstrated by a complete lack of clot lysis on TEG. The receiver operating characteristic curve to predict venous thromboembolism (VTE) was only significant for Lys-30. Likewise, Pavoni and colleagues, performing a ROTEM analysis, confirmed that patients with severe COVID-19 pneumonia had a hypercoagulation state that persisted over time [16]. Forty patients demonstrated acceleration of the propagation phase of blood clot formation (reduction of CFT in the INTEM and EXTEM) and a significantly higher clot strength (increase in MCF in the INTEM, EXTEM and FIBTEM) as compared to the normal range limits; this hypercoagulable state persists in the first days, but it decreases over time. Alike, Mortus et al. enrolled 21 critically ill COVID-19 patients and reported that 19 of them presented with hypercoagulable TEG, defined as elevated fibrinogen activity greater than a 73° angle or MA more than 65mm on TEG with heparinase correction [17]. Innate TEG MA was significantly greater for the high thrombotic event rate group (≥2 thrombotic events) than the low event rate group (0-1 thrombotic events), while elevated MA was observed in 10 patients (100%) in the high thrombotic event rate group vs. five patients (45%) in the low event rate group. No abnormal fibrinolytic activity was noted. In the same way, nineteen ICU COVID-19 patients were recruited by Ibanez et al. and demonstrated clot firmness above the normal range in the EXTEM and FIBTEM tests, while clot lysis was decreased [18]. Clot firmness and clot lysis were assessed against the normal range and reported levels in the healthy controls, respectively. COVID-19 patients showed a hypercoagulable state with a thromboelastometry pattern mainly characterized by decreased fibrinolytic capacity. Moreover, there was no significant correlation between the ROTEM parameters and the Sequential Organ Failure Assessment (SOFA) score. In another recent study, all hospitalized COVID-19 patients showed elevated values of EXTEM-MCF and FIBTEM-MCF at admission to the hospital, also suggesting a hypercoagulable state, and this pattern became more pronounced in patients with the more severe disease [19]. Specifically, the authors showed that all COVID-19 patients (both with mild and severe COVID-19 pneumonia) had significantly longer CT, increased MCF and shorter CFT, as compared with healthy controls, and that severely ill subjects had longer CT, increased MCF and shorter CFT compared with subjects with mild infections. Furthermore, based on the modified TEM tissue plasminogen activator (tPA) LI30 variable, ICU COVID-19 patients demonstrated a much less effective fibrinolysis as related with the normal controls, while the LI30 of ICU patients with thrombosis was significantly higher than other ICU COVID-19 patients with similar disease severity [20]. Moreover, Blasi et al. found ROTEM parameters in 23 COVID-19 patients admitted both to the ICU or to the general ward to be largely within the normal ranges, except for elevated MCF in part of the patients [21], while supranormal clot firmness and minimal fibrinolysis were the key findings in six patients with COVID-19-associated respiratory failure admitted to the ICU [22]. Of note, this hypercoagulable state was not appreciable on conventional tests of coagulation. Among 21 ICU COVID-19 patients, half of them met the criteria for fibrinolysis shutdown, defined as having an EXTEM maximum lysis of <3.5%, while eight of nine of the VTE patients exhibited fibrinolysis shutdown [23]. Finally, Hoechter et al. reported higher FIBTEM MCF in COVID-19 compared to non-COVID-19 ICU patients, suggesting a higher procoagulatory potential in the case of COVID-19 critical illness [24]. All the aforementioned studies support that COVID-19 patients with acute respiratory failure present a severe hypercoagulability rather than a consumptive coagulopathy, often associated with fibrinolysis shutdown. The mismatched D-dimer increments might be due to the augmentation of local fibrinolysis in alveoli by urokinase-type plasminogen activator (u-PA) released from alveolar macrophages [25]. It is noteworthy that critically ill COVID-19 patients demonstrated a more hypercoagulable and hypofibrinolytic profile related to those with COVID-19 mild illness, while hypercoagulability and hypofibrinolysis were evident in both patient groups, as compared to healthy controls [19]. This indicates that hypercoagulability in COVID-19 infection might be associated with disease severity. However, a correlation between ROTEM parameters and severity illness scores has not been reported [18]. It should be also pointed out that, in most cases, ICU COVID-19 patients have longer or normal CT/R values [12,13,17,18,19]. This is the only parameter not supporting the procoagulant profile of critically ill COVID-19 patients. Treatment with low molecular weight heparin (LMWH)/unfractionated heparin in prophylactic doses or the functional “exhaustion” of coagulation factors might account for this find. This is in keeping with the normal endogenous thrombin potential (ETP) levels reported in ICU COVID-19 patients on prophylactic anticoagulation [20]. Finally, some authors reported a more thrombogenic profile in critically ill COVID-19 patients with thrombotic complications compared to COVID-19 patients without thrombosis [15,17,20,23]. However, the great variability in definition of both the procoagulant profile and the clinical outcome assessment, in parallel with the small sample sizes in most of the studies, does not allow the establishment of a clear association between the hypercoagulable state and thrombotic events. 

## 4. Coagulation Disorders as Detected by VMs in Critical Illness

Despite the ongoing debate regarding the degree that COVID-19 behaves differently than other well-studied critical illnesses, it is probably reasonable to assume that COVID-19 affects the pathways of inflammation and coagulation perhaps in a different scale and time frame. Therefore, established laboratory markers of deregulated inflammation and coagulation are unsurprisingly affected by this new infectious disease.

The main interactions between coagulation and inflammation involve the procoagulant, anticoagulant and fibrinolytic pathways. Regarding adult sepsis, in most relevant studies, VMs showed sepsis-induced coagulopathy, but changes in coagulation parameters were heterogeneous, with patients showing both hyper- and hypocoagulability [3]. Variability in study designs, populations and timing of measurements, differences in disease severity and lack of clearness in defining coagulopathy probably account for this inconsistency. Inflammation-induced coagulopathy is a very dynamic process, ranging from the initial hypercoagulability towards a subsequent hypocoagulable profile, depending on the critical illness evolvement [25]. The initial host inflammatory response to an invading organism usually results in a procoagulant phase in order to limit the spread of the pathogens [26], but the consumption of clotting factors due to ongoing thrombosis finally leads to a hypocoagulable phase. The development of a hypocoagulable state is consistent with the pathophysiology of “consumption coagulopathy” during DIC, in which microvascular thrombi are formed at the expense of a bleeding tendency because of low levels of platelets and coagulation factors [27]. Interestingly, the degree of hypocoagulation has been found to be associated with DIC and the severity of organ failure [28,29,30,31]. 

However, this sequence of events is not always the case. For instance, using VMs, a clear hypocoagulable profile was noted at the early phase of neonatal sepsis [32,33] associated with disease severity [32]. The low levels of the vitamin K-dependent procoagulant factors, contact factors and increased fibrinolytic activity at birth might account for the disruption of the subtle hemostatic balance, predisposing ill neonates to develop hypocoagulability in the initial phase of critical illness [34].

Accordingly, the pathophysiology for COVID-19-related systemic microthrombosis seems to be specific and, in particular, different from DIC [1]. In severe COVID-19 infections, the enhanced cytokine release (cytokine storm) during virus infection seems to stimulate an overwhelming procoagulant reaction, leading to a situation in which coagulation itself contributes to the worsening of the disease severity, with widespread microvascular thromboses, subsequent organ dysfunctions and worse clinical outcomes before the development of consumptive coagulopathy arises [4,7].

In-line with fibrinolysis shutdown, often reported in COVID-19 critically ill patients, hypofibrinolysis has also been demonstrated in several studies in adult sepsis [25,27,35,36] and neonatal sepsis [37], but the clinical relevance of this finding has not been determined. In some cases, thromboelastometrical lysis indexes (LIs) proved to be reliable biomarkers for both the early identification [25,35] and prognosis [36] of patients suffering from sepsis. However, it should be stressed that fibrinolysis impairment in ICU COVID-19 patients has not been a consistent finding, even among studies using the same viscoelastic method [13,14,15,18]. Contradictory findings have also previously been reported on VMs’ diagnostic capacity to detect abnormal fibrinolysis [38,39,40], while concerns have been raised about the sensitivity of VMs for identifying fibrinolysis in all patients [41]. An important limitation of all the studies published in the field, probably accounting for the discrepant findings, is the absence of a single gold standard assay for the global measurement of fibrinolytic activity and, consequently, the different definitions of hyperfibrinolysis, as detected by VMs used in various studies. Finally, different ROTEM and TEG performances in assessing the fibrinolytic capacity in patients with sepsis, disease severity and variable timing of sampling might contribute to the discrepancies regarding fibrinolysis in ICU COVID-19 patients [18].

Based on these data, it can be assumed that the type of coagulopathy and the relevant clinical symptoms appearing, due to the interaction between inflammation and coagulation, are closely dependent on the intensity and duration of inflammatory stimuli, the previous hemostatic balance and the timing of the measurements. VMs have the capacity of assessing this delicate equilibrium between procoagulant, anticoagulant and fibrinolytic factors, revealing the coagulation disturbances that might lead either to bleeding or thrombotic disorders. However, they are considered inappropriate to assess each hemostatic component individually and independently [37]. Based on this conception, we have to examine the role of VMs in clinical practice regarding coagulopathy in critical illness. 

## 5. Clinical Utility of VMs in Assessment of Coagulopathy in Critically Ill COVID-19 Patients

The following principle has to be considered when performing VMs: Thromboelastometric tests are not designed to predict thrombotic or bleeding events but only to help determine the cause of bleeding [42]. Thus, beyond their use in transfusion algorithms, considering VMs’ clinical utility in other clinical settings, like critical illnesses, several essential issues should be taken into account. No universal definitions exist of hypo- and hypercoagulability. Some authors define hypo- or hypercoagulable states by measurements lying outside preset reference ranges; others compare patients with healthy individuals, and some compare mean or median values among or within different patient groups. To compare patient groups and possibly assess therapeutic interventions involving the hemostatic system, validated reference values and definitions are critical [3]. Moreover, the hemostatic profile of a critically ill patient usually changes substantially over time; thus, timing may greatly influence the TEG/ROTEM results. Finally, VMs cannot assess each hemostatic component separately. 

All these limitations are also in place regarding VM measurements in the severe COVID-19 infection. Characteristically, in almost all relevant studies, different criteria were used to define the hypercoagulability. Furthermore, the corresponding parameters of each assay probably were not equally affected by the administration of several anticoagulant treatments. Thus, these results are difficult to be standardized and used in order to “quantify” the hypercoagulability and define the prognosis or optimal treatment strategy. Given the background, and keeping in mind that VMs provide overall information on coagulation and fibrinolysis equilibrium, it would rather not be a prudent approach to apply an antiplatelet or fibrinolytic or enhanced anticoagulant therapy to treat hypercoagulability, based exclusively on the VM findings and without the use of specific assays to evaluate certain hemostatic components. 

## 6. Conclusions

Considering these characteristics and limitations of VMs, the results of these assays should be evaluated in a particular context. They can effectively provide insight into the pathophysiology of coagulopathy, detecting the presence of hypercoagulability in critically ill COVID-19 patients. However, the type and degree of coagulopathy might be inadequate in order to predict the outcome, establish a diagnosis or guide an antigoagulant therapy. To achieve these goals, much more needs to be done. Studies with adequate sample sizes to establish reference ranges for each viscoelastic test or multicenter study properly designed to define the common cut-off values of hypo- and hypercoagulability or threshold values to predict prognosis are necessary. Finally, regarding the utility of VMs in order to guide anticoagulant, antiplatelet or fibrinolytic therapy, the parallel use of other specific assays evaluating the hemostatic components affected by the corresponding treatment, in association with the clinical outcome, should be considered a prerequisite before their application in clinical practice. 

## Figures and Tables

**Table 1:** Studies assessing the hemostatic profiles in critically ill COVID-19 patients by viscoelastic methods.

Study	Patients (n)	Viscoelastic Assay	Variables	Controls	Findings	Treatment	Clinical Outcome
Ranucci et al., 2020 [12]	COVID 19 ICU ARDS (16)	STA-NeoPTimal (ultrasound-based technology)	CoT, CS, FCS, PCS	The upper limit of the normal reference ranges	Procoagulant profile	Τhromboprophylaxis	No major VTE
Ranucci et al., 2020 [12]	COVID 19 ICU ARDS (10)	STA-NeoPTimal (ultrasound-based technology)	CoT, CS, FCS, PCS		Progression toward normalization	Increased thromboprophylaxis	
Panigada et al., 2020 [13]	COVID-19 ICU (24)	TEG	R, K, K angle, MA, Lys-30	Reference ranges, previously established in 40 healthy adult subjects	Hypercoagulability, hyperfibrinolysis	Thromboprophylaxis	23% developed in-hospital VTE
Spiezia et al., 2020 [14]	COVID-19 ICU ARDS (22)	ROTEM (EXTEM, INTEM, FIBTEM)	CT, CFT, MCF, ML	44 healthy subjects	Hypercoagulability	Thromboprophylaxis	Patients with fibrinolysis shutdown had a 40% rate of VTE compared to 5% in patients without shutdown
Wright et al., 2020 [15]	COVID-19 ICU (44)	TEG/TEG heparinase	R, K, K angle, MA, Lys-30	Reference ranges	Hypercoagulability, fibrinolysis shutdown	Thromboprophylaxis	
Pavoni et al., 2020 [16]	COVID-19 ICU, severe pneumonia (40)	ROTEM (EXTEM, INTEM, FIBTEM)	CT, CFT, MCF, ML	Reference ranges	Hypercoagulability	Thromboprophylaxis	50% developed thrombotic event
Mortus et al., 2020 [17]	COVID-19 ICU (21)	TEG /TEG heparinase	R, K, K angle, MA, Lys-30	Reference ranges	Hypercoagulability	Thromboprophylaxis or therapeutic anticoagulation	62% developed thrombotic events. MA was significantly greaterfor the high thrombotic event rate group than the low event rate group.
Ibanez et al., 2020 [18]	COVID-19 ICU (19)	ROTEM (EXTEM, INTEM, FIBTEM)	CT, CFT, MCF, LI30, LI60	Reference ranges	Hypercoagulability, hypofibrinolysis	Thromboprophylaxis	26% had thrombotic complications, 10% had bleeding
Almskog et al., 2020 [19]	COVID-19 mild (40) and severe pneumonia (20)	ROTEM (EXTEM, FIBTEM)	CT, CFT, MCF.	89 healthy individuals from a previously published study	Hypercoagulability in hospitalized patients with mild to severe COVID-19 pneumonia compared with healthy controls	Low prophylactic dose; High prophylactic dose; Treatment dose	
Nougier et al., 2020 [20]	COVID-19 ICU (19), COVID-19 mild infection (4)	tPA-modified ROTEM	MCF, an angle, LI30	10 healthy volunteers	Hypercoagulability and hypofibrinolysis in COVID-19 patients with thrombosis.	Thromboprophylaxis	COVID-19 ICU patients had 12.5% thrombotic complications
Blasi et al., 2020 [21]	COVID-19 ICU (12), COVID-19 mild infection, (11)	ROTEM (EXTEM, INTEM, FIBTEM)	CT, MCF, LI60	Reference ranges	Hypercoagulability in part of the patients	Normal to intensified anticoagulant therapy	Three COVID-19 ICU patients had thrombotic complications:
Collett et al., 2020 [22]	COVID-19 ICU (6)	ROTEM (EXTEM, INTEM, FIBTEM)	A10, CFT, MCF, ML	Reference ranges	Hypercoagulability with minimal fibrinolysis	Thromboprophylaxis	Three patients developed VTE
Creel-Bulos et al., 2020 [23]	COVID-19 ICU (21)	ROTEM (EXTEM, FIBTEM)	CT, MCF, ML	Reference ranges	Fibrinolysis shutdown	Thromboprophylaxis	Nine patients (42.9%) had VTE. 73% (*n* = 8) were diagnosed with fibrinolytic shutdown
Hoechter et al., 2020 [24]	COVID-19 ICU (11), Non-COVID-19 ICU (14)	ROTEM (EXTEM, FIBTEM)	CT, CFT, MCF, ML	Reference ranges	COVID-19 patients presented with higher coagulatory potential	Thromboprophylaxis	

Abbreviations: ARDS, acute respiratory distress syndrome, CFT, clot formation time, CT, clotting time, CoT, coagulation time, CS, clot stiffness, FCS, fibrinogen contribution to CS, ICU, intensive care unit, K defines the velocity of the clot formation, LMWH, low molecular weight heparin, MA, maximal amplitude, MCF, maximum clot firmness, ML, maximum lysis, LI30, lysis index at 30 min, LI60, lysis index at 60 min, Lys-30, the percentage decrease of the clot amplitude at 30 min, PCS, platelet contribution to CS, R, measure of the clotting time, ROTEM, rotational thromboelastometry, TEG, thromboelastography, tPA, tissue plasminogen activator and VTE, venous thromboembolic events.
